# Differential Effects of Carboplatin, Metformin, and Boric Acid on Cell Viability, Migration, and Oxidative Stress in MCF-7 Breast Cancer and CRL-4010 Normal Mammary Epithelial Cells

**DOI:** 10.3390/biomedicines14092073

**Published:** 2026-09-15

**Authors:** Nilüfer Ece Süren, Burcu Biltekin, Sevgin Degirmencioglu, Hafize Uzun, Ayhan Bilir

**Affiliations:** 1 Department of Histology and Embryology, Faculty of Medicine, Istanbul Atlas University, Istanbul 34403, Turkey; nlfrece.suren@gmail.com (N.E.S.); ayhan.bilir@atlas.edu.tr (A.B.); 2 Department of Biochemistry, Faculty of Medicine, Kirklareli University, Kirklareli 39060, Turkey; sevgindegirmencioglu@klu.edu.tr; 3 Department of Biochemistry, Faculty of Medicine, Istanbul Atlas University, Istanbul 34408, Turkey; hafize.uzun@atlas.edu.tr

**Keywords:** breast cancer, carboplatin, metformin, boric acid, oxidative stress, superoxide dismutase, cell viability, wound-healing assay, MCF-7

## Abstract

**Background/Objectives:** Oxidative stress is a key contributor to breast cancer (BC) progression and therapeutic response. Although carboplatin is widely used in BC treatment, the effects of combining carboplatin with metformin and boric acid (BA) on cellular redox homeostasis remain poorly understood. This study investigated the effects of these agents on cell viability, migration, and oxidative stress in BC and normal mammary epithelial cells. **Methods:** Human BC MCF-7 cells and normal mammary epithelial CRL-4010 cells were treated with carboplatin, metformin, BA, and selected combination regimens. Cell viability was assessed using the Cell Counting Kit-8 (CCK-8) assay following 24, 48, and 72 h of treatment. Cell migration was evaluated using a scratch wound-healing assay, and intracellular oxidative stress responses were assessed by measuring superoxide dismutase (SOD) activity. Statistical analyses were performed using one-way ANOVA followed by Fisher’s least significant difference (LSD) post hoc test. **Results:** Treatment responses were both time- and treatment-dependent in MCF-7 and CRL-4010 cells. Combination regimens significantly reduced cell viability after 48 and 72 h (all *p* < 0.0001) and generally exhibited greater inhibitory effects than single-agent treatments. Metformin- and BA-containing combinations also produced more pronounced inhibition of cell migration. Significant treatment-related alterations in SOD activity were observed in both cell lines, indicating treatment-associated changes in antioxidant enzyme activity. While several combination regimens decreased SOD activity, others induced increased SOD activity, suggesting differential oxidative stress responses between malignant and non-malignant cells. **Conclusions:** Carboplatin, metformin, and BA significantly modulated cell viability, migration, and antioxidant responses in both BC and normal mammary epithelial cells. Combination treatments generally produced greater inhibitory effects on cell viability and migration than selected single-agent treatments, particularly in MCF-7 cells. These findings demonstrate treatment-dependent changes in cell viability, migration, and SOD activity and provide a basis for further investigation of the molecular mechanisms underlying carboplatin-based combination treatments.

## 1. Introduction

Breast cancer (BC) remains the most frequently diagnosed malignancy among women worldwide and was responsible for approximately 694,000 deaths in 2022 [1,2]. Based on the expression of estrogen receptor (ER), progesterone receptor (PR), and human epidermal growth factor receptor 2 (HER2), BC is classified into four major molecular subtypes: Luminal A, Luminal B, HER2-positive, and triple-negative BC (TNBC) [3]. Despite advances in diagnosis and treatment, BC continues to pose a major global health challenge.

Current BC management includes surgery, radiotherapy, chemotherapy, endocrine therapy, and targeted therapies. Treatment selection is guided by clinicopathological characteristics such as tumor subtype, tumor size, lymph node involvement, histological grade, patient age, and receptor status [4,5,6]. Chemotherapy remains a cornerstone of treatment, particularly in advanced and high-risk disease [7,8].

Oxidative stress results from an imbalance between reactive oxygen species (ROS) generation and antioxidant defense systems [9]. Accumulating evidence indicates that oxidative stress contributes to cancer initiation, progression, metastasis, and therapeutic response. Elevated ROS levels have been associated with genomic instability, tumor heterogeneity, aggressive tumor behavior, and poor clinical outcomes [10,11]. Therefore, targeting oxidative stress pathways has emerged as a promising approach in cancer therapy.

Carboplatin is a widely used platinum-based chemotherapeutic agent whose antitumor activity is primarily mediated through DNA crosslink formation and apoptosis induction [12,13,14]. In addition, carboplatin can increase intracellular ROS production, contributing to oxidative damage and cancer cell death [12].

Metformin, a first-line therapy for type 2 diabetes mellitus, has attracted considerable attention because of its potential anticancer effects [15]. Experimental studies have suggested that metformin may affect tumor cell growth through alterations in cellular metabolism, proliferation, and redox-related processes. However, the specific molecular pathways underlying these effects may vary according to cellular context and treatment conditions [16,17].

Boric acid (BA), a naturally occurring boron compound, exhibits antioxidant, anti-inflammatory, and antiproliferative properties. Previous studies have demonstrated that BA may reduce oxidative stress and suppress proliferation in several cancer cell lines, including BC cells [18,19,20,21].

Despite growing evidence regarding the anticancer properties of carboplatin, metformin, and BA, their comparative effects on oxidative stress in BC and normal mammary epithelial cells remain unclear. Therefore, this study aimed to investigate the effects of these agents on oxidative stress in human BC (MCF-7) and normal mammary epithelial (CRL-4010) cells. Following determination of optimal treatment concentrations using CCK-8 assays, wound-healing and superoxide dismutase (SOD) analyses were performed to evaluate treatment-induced changes in cellular behavior and oxidative stress status.

## 2. Materials and Methods

**Figure 1:**
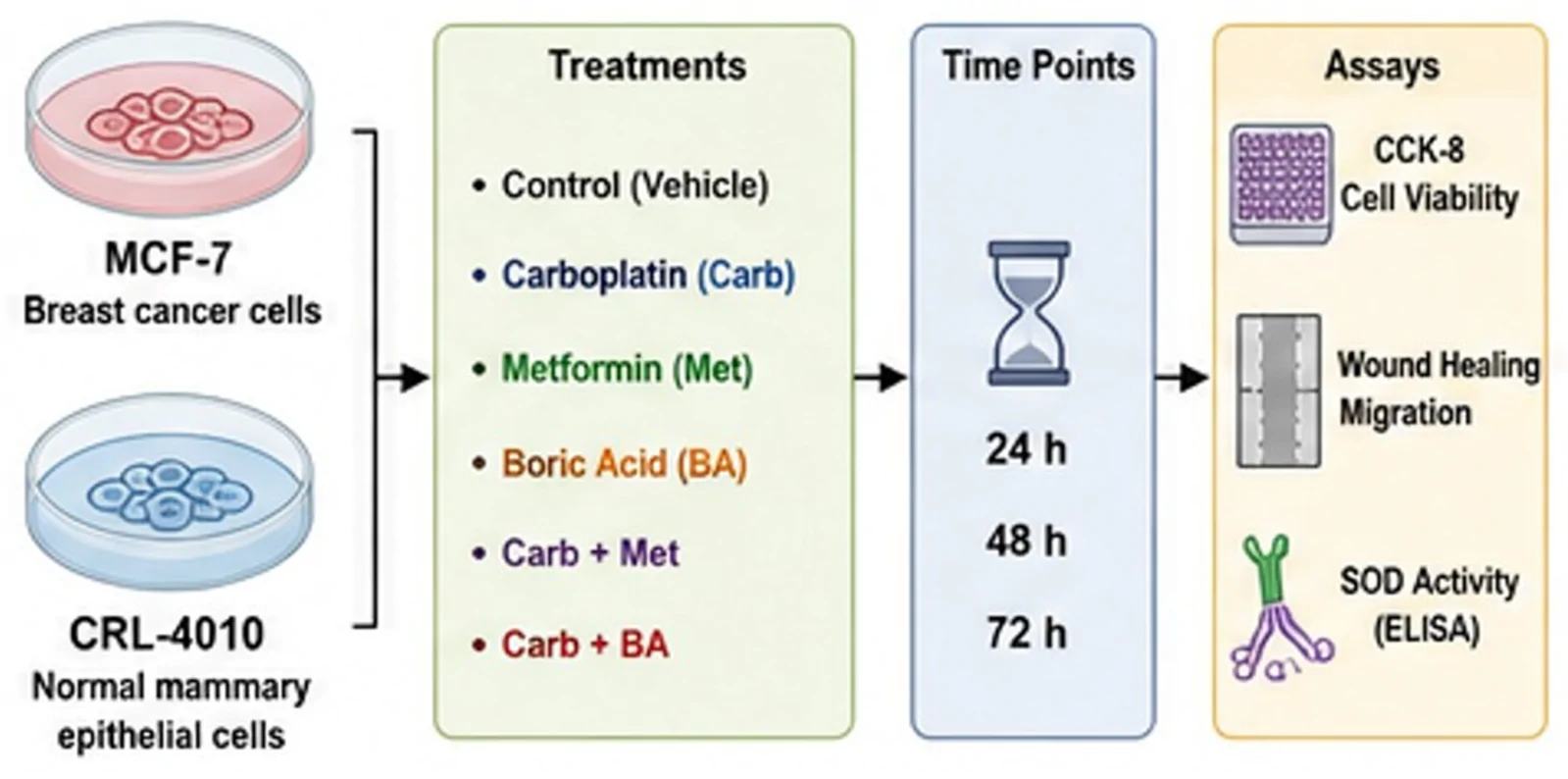
Experimental workflow of the study.

Human BC MCF-7 cells and normal mammary epithelial CRL-4010 cells were treated with carboplatin, metformin, BA, and selected drug combinations. Cell viability was assessed using the Cell Counting Kit-8 (CCK-8) (Abbkine, Wuhan, China) assay after 24, 48, and 72 h of treatment. Cell migration was evaluated by scratch wound-healing assay, and oxidative stress status was assessed by measuring intracellular SOD activity. The experimental design and analytical workflow are illustrated in Figure 1.

### 2.1. Drug Nomenclature and Preparation

In this study, carboplatin (cis-diammine(1,1-cyclobutanedicarboxylato)platinum(II), a second-generation platinum-based chemotherapeutic agent; Onko Drug, Istanbul, Türkiye), metformin hydrochloride (1,1-dimethylbiguanide hydrochloride, a biguanide antidiabetic agent; Cayman Chemical, Ann Arbor, MI, USA), and BA (H_3_BO_3_, a naturally occurring boron-containing compound; Sigma-Aldrich, St. Louis, MO, USA) were evaluated individually and in combination for their effects on BC and normal mammary epithelial cells.

Preparation of carboplatin: Carboplatin was freshly prepared in Dulbecco’s Modified Eagle Medium (DMEM) immediately before each experiment to ensure optimal stability and reproducibility.

Preparation of metformin: Metformin hydrochloride was dissolved in DMEM to prepare a stock solution, which was stored at 4 °C until use. Working concentrations were freshly prepared by dilution in culture medium immediately prior to treatment.

Preparation of BA: BA was dissolved in DMEM to prepare a stock solution and stored at 4 °C. Appropriate working concentrations were prepared immediately before application to the cells.

For all experiments, treatment solutions were prepared in complete culture medium and administered at the indicated concentrations. Control cells received culture medium alone under identical experimental conditions.

### 2.2. Cell Lines and Culture Conditions

Human breast adenocarcinoma MCF-7 (ATCC, HTB-22) cells and normal mammary epithelial CRL-4010 (ATCC, hTERT-HME1 [ME16C]) cells were used throughout the study.

Cells were cultured in Dulbecco’s Modified Eagle Medium (DMEM) supplemented with 10% heat-inactivated fetal bovine serum (FBS), 100 IU/mL penicillin, and 100 μg/mL streptomycin. Cultures were maintained at 37 °C in a humidified incubator containing 5% CO_2_.

Frozen cell stocks stored in liquid nitrogen were rapidly thawed and seeded into T25 culture flasks containing complete growth medium. After 24 h, the culture medium was replaced to remove residual dimethyl sulfoxide (DMSO). Cells were routinely monitored and passaged upon reaching 80–90% confluence.

### 2.3. Determination of Treatment Concentrations and Exposure Times

Preliminary dose- and time-response experiments were performed to guide the selection of treatment concentrations and exposure times. The concentrations used in the main experiments were selected based on these preliminary experiments together with concentration ranges previously reported in in vitro studies. Following cell counting using a hemocytometer, 1 × 10^4^ cells/well were seeded into 96-well culture plates containing 200 μL of complete medium and incubated for 24 h.

For both cell lines, experimental groups consisted of Control, Carboplatin, Metformin, BA, Carboplatin + Metformin, Carboplatin + BA, and BA + Metformin. Carboplatin was tested at 10 and 50 μM, metformin at 25 and 40 mM, and BA at 10–50 μM. These concentrations were selected to provide measurable treatment responses across the experimental period while avoiding complete loss of cell viability. Following treatment, cells were incubated for 24, 48, or 72 h before further analyses.

### 2.4. Cell Viability Assay

Cell viability was assessed using the Cell Counting Kit-8 (CCK-8; GlpBio, Montclair, CA, USA) according to the manufacturer’s instructions. After 24, 48, and 72 h of treatment, 10 μL of CCK-8 reagent was added to each well and the plates were incubated for 3 h at 37 °C.

Absorbance was measured at 450 nm using a microplate reader (Bio-Tek, Winooski, VT, USA). Cell viability was expressed relative to untreated control cells.

### 2.5. In Vitro Scratch Wound-Healing Assay

For wound-healing experiments, cells were seeded into six-well plates at a density of 6 × 10^5^ cells/well in 2 mL of complete medium and allowed to attach for 24 h. Upon reaching approximately 80% confluence, a linear scratch was created using a sterile pipette tip. Detached cells were removed by washing with phosphate-buffered saline (PBS).

Cells were subsequently treated with the selected concentrations of carboplatin, metformin, BA, or their combinations. The time of treatment initiation was designated as 0 h. Cells were incubated under standard culture conditions and harvested after 24, 48, and 72 h.

### 2.6. Morphological Evaluation

Cell morphology was evaluated by light microscopy before treatment and at the designated experimental endpoints. Representative images were captured to assess treatment-induced morphological alterations and were compared among experimental groups.

### 2.7. Determination of Superoxide Dismutase (SOD) Activity

SOD activity levels were determined in both control and treatment groups using a CheKine™ Micro Superoxide Dismutase (SOD) Activity Assay Kit (Abbkine Scientific Co., Ltd., Wuhan, China) according to the manufacturer’s instructions. Briefly, cell lysates were prepared from harvested cells and incubated with the assay reagents provided in the kit. SOD activity was quantified based on the enzyme-mediated inhibition of superoxide anion-dependent reactions using a colorimetric method. Absorbance was measured at 450 nm using a microplate reader, and SOD activity levels were calculated according to the manufacturer’s protocol. All measurements were performed in triplicate, and the results were expressed as mean ± standard deviation (SD).

### 2.8. Statistical Analysis

Statistical analyses were performed using GraphPad Prism version 9.0 (GraphPad Software, San Diego, CA, USA). Data are presented as mean ± standard deviation (SD) from at least three independent experiments. Comparisons among multiple groups were performed using one-way analysis of variance (ANOVA), followed by Dunnett’s multiple-comparisons test for comparisons of treatment groups with the untreated control. The multiple-comparison procedure was used to control the family wise error rate. All statistical tests were two-sided, and adjusted *p*-values < 0.05 were considered statistically significant.

## 3. Results

### 3.1. Experimental Design

Figure 1 illustrates the overall experimental workflow of the study. Human BC MCF-7 cells and normal mammary epithelial CRL-4010 cells were treated with carboplatin, metformin, BA, and selected combination regimens. Cell viability, migratory capacity, and oxidative stress responses were subsequently evaluated using CCK-8, wound-healing, and superoxide dismutase (SOD) activity assays, respectively.

### 3.2. Effects of Carboplatin, Metformin, and Boric Acid on Cell Viability

**Figure 2:**
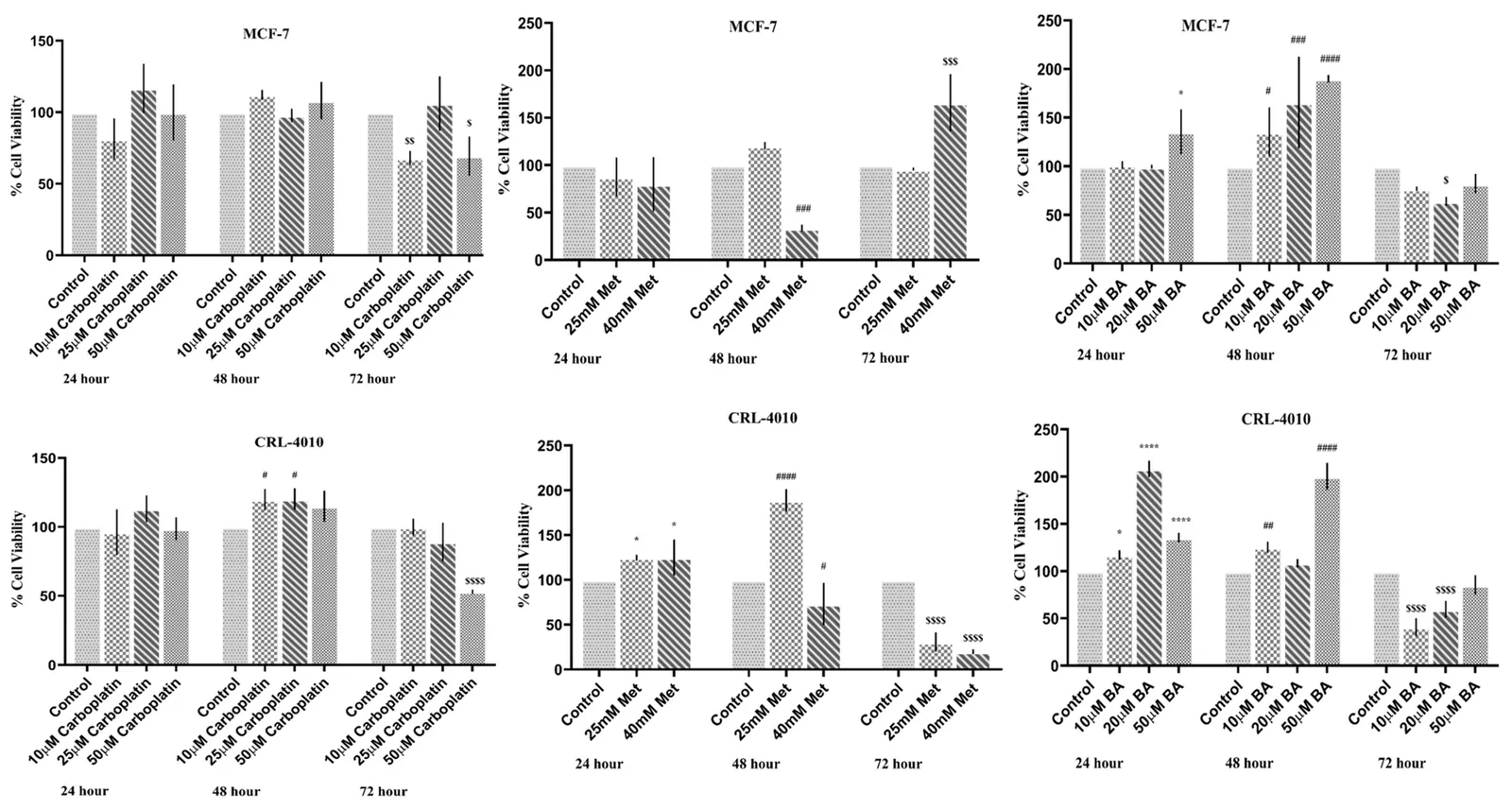
Cytotoxic effects of individual treatments of carboplatin, metformin, and boric acid on MCF-7 breast cancer cells and CRL- 4010 normal mammary epithelial cells. Cell viability was evaluated using the Cell Counting Kit-8 (CCK-8) assay after 24 h, 48 h, and 72 h of treatment. Results are expressed as the percentage of the untreated control (100%) and presented as the mean ± standard deviation (SD) of three independent experiments. Statistical significance was determined using one-way analysis of variance (ANOVA) followed by Fisher’s least significant difference (LSD) post hoc test. (* *p* < 0.05, **** *p* < 0.0001; # *p* < 0.05, ## *p* < 0.01, ### *p* < 0.001, #### *p* < 0.0001; ^$^ *p* < 0.05, ^$$^ *p* < 0.01, ^$$$^ *p* < 0.001, ^$$$$^ *p* < 0.0001).

**Figure 3:**
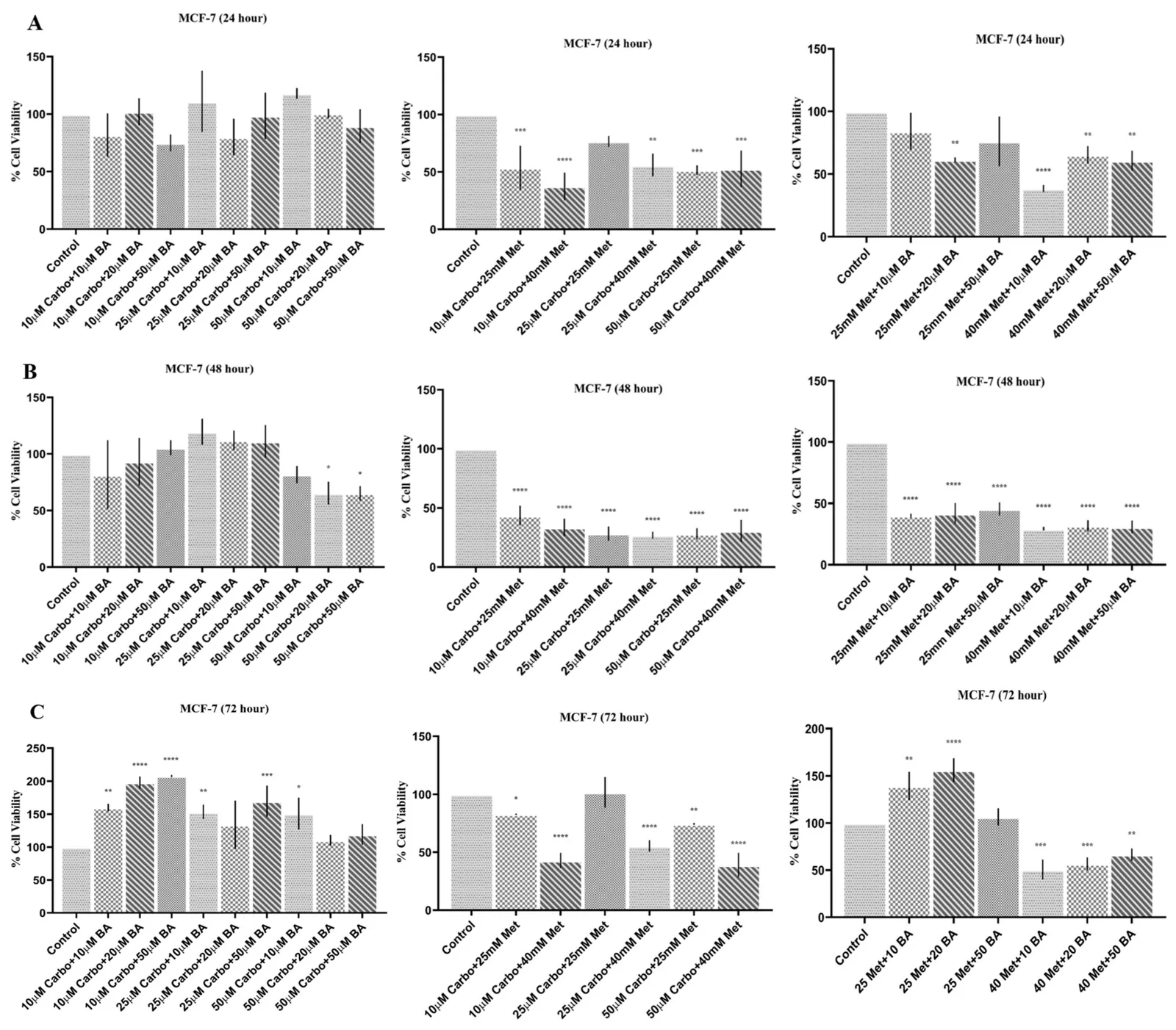
Cytotoxic profiles of dual combinations involving carboplatin, boric acid, and metformin on MCF-7 breast cancer cells. Cell viability was evaluated using the CCK-8 assay following 24 h (**A**), 48 h (**B**), and 72 h (**C**) of exposure to specified drug combinations. Untreated cells served as the control group (100% viability). Data are expressed as mean ± standard deviation (SD) from three independent experiments. Statistically significant differences compared to the control group are indicated by asterisks: * *p* < 0.05, ** *p* < 0.01, *** *p* < 0.001, and **** *p* < 0.0001.

The effects of carboplatin (Carbo), metformin (Met), boric acid (BA), and their combinations on MCF-7 cell viability after 24, 48, and 72 h of treatment are presented in Figure 2 and Figure 3. After 24 h, monotherapies of Carbo (10–50 µM) and Met (25–40 mM) did not alter cell viability (p>0.05), whereas high-dose BA (50 µM) induced a significant reduction (p=0.0153). Co-treatments of Carbo+Met and Met+BA generally decreased viability significantly, particularly in combinations featuring 40 mM Met (p<0.005), while all Carbo+BA combinations remained ineffective (p>0.05). At 48 h, Carbo alone sustained no cytotoxic effect, but Met (25–40 mM) and BA (20–50 μM) monotherapies significantly impaired viability (p<0.05). Notably, enhanced or additive cytotoxicity became prominent in dual therapies; all Carbo+Met and Met+BA combinations exerted potent cytotoxic effects (p<0.0001), whereas Carbo+BA showed significant activity only at the highest Carbo dose (50 μM, p<0.05). Following 72 h of treatment, prolonged incubation revealed significant cytotoxicity for BA across all tested doses (p<0.05). Carbo and Met monotherapies exhibited selective efficacy, showing significant activity primarily at specific concentrations (10 and 50 μM for Carbo; 40 mM for Met; p<0.05). Except for a few specific doses, almost all combined treatment regimens significantly suppressed MCF-7 cell viability compared to the control group.

**Figure 4:**
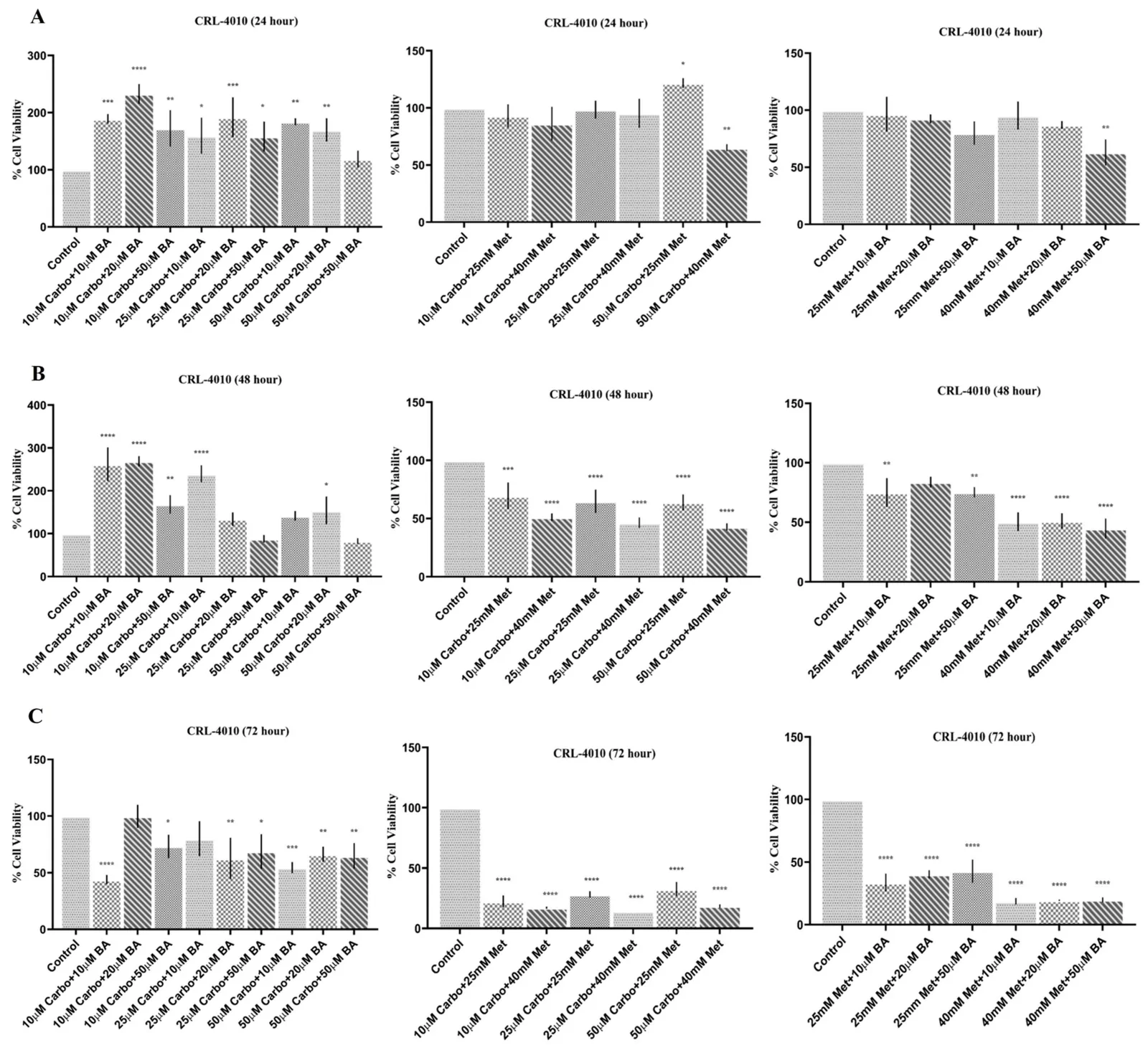
Cytotoxic profiles of dual combinations involving carboplatin, boric acid, and metformin on CRL-4010 cells. Cell viability was evaluated using the CCK-8 assay following 24 h (**A**), 48 h (**B**), and 72 h (**C**) of exposure to specified drug combinations. Untreated cells served as the control group (100% viability). Data are expressed as mean ± standard deviation (SD) from three independent experiments. Statistically significant differences compared to the control group are indicated by asterisks: * *p* < 0.05, ** *p* < 0.01, *** *p* < 0.001, and **** *p* < 0.0001.

The effects of carboplatin (Carbo), metformin (Met), boric acid (BA), and their combinations on CRL-4010 cell viability after 24, 48, and 72 h of treatment are presented in Figure 2, Figure 3 and Figure 4. After 24 h of treatment, BA monotherapy rapidly reduced CRL-4010 viability across all concentrations (10–50 μM, p<0.05), while Carbo and Met alone had no effect. Reflecting this BA sensitivity, nearly all Carbo+BA combinations significantly decreased cell viability (p<0.05), whereas Carbo+Met combinations were largely ineffective except when paired with 50 μM Carbo (p<0.05). Following 48 h of incubation, Met (25 mM) and BA (10 and 50 μM) significantly reduced cell survival (p<0.05). Combined treatments demonstrated enhanced efficacy, as all Carbo+Met combinations significantly reduced viability (p<0.0004), and Met+BA combinations followed a similar trend (p<0.005, except 25 mM Met + 20 μM BA). Carbo+BA combinations showed selective cytotoxicity, primarily driven by the lower Carbo dose (10 μM, p<0.005). After 72 h, Carbo showed significant cytotoxicity only at 50 μM (p=0.0003), whereas Met induced robust, concentration-independent cytotoxicity (p<0.0001). BA maintained significant activity at 10 and 20 μM (p<0.005). Crucially, both Carbo+Met and Met+BA combinations consistently achieved highly significant reductions in cell viability across all tested combinations (p<0.0001).

### 3.3. Wound-Healing Assay

**Figure 5:**
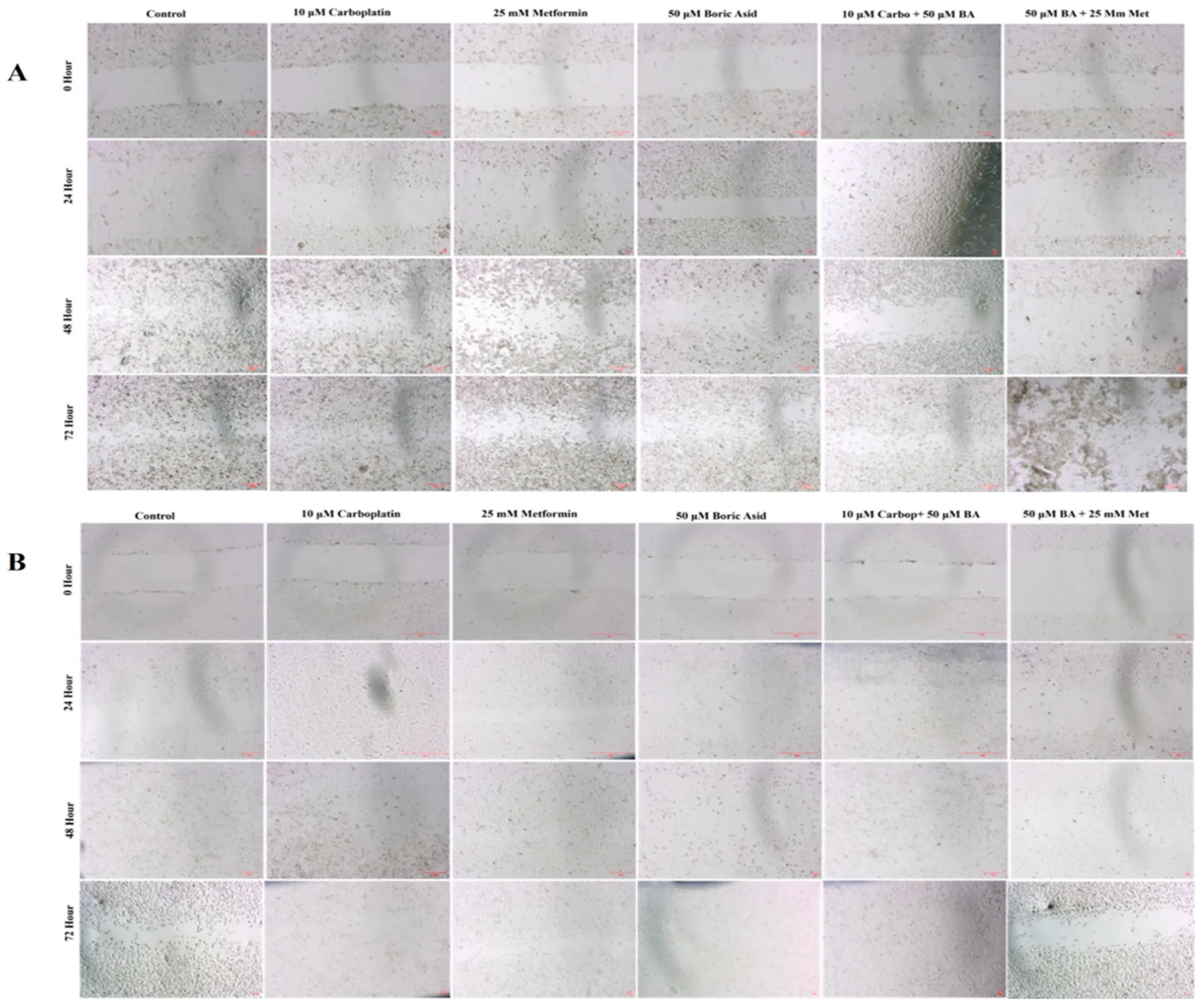
Representative wound-healing assay images illustrating the effects of carboplatin (10 μM), metformin (25 mM), boric acid (50 μM), and their combinations on MCF-7 (**A**) and CRL-4010 (**B**) cell migration. Confluent monolayers were scratched to generate a uniform wound, followed by treatment with the indicated compounds. Images were captured at 0, 24, 48, and 72 h to evaluate wound closure over time. Scale bar = 100 μM.

The migratory capacity and wound closure dynamics of MCF-7 cells were evaluated over 72 h (Figure 5A). The control group, along with 10 µM Carbo, 25 mM Met, and 50 µM BA monotherapies, showed significant time-dependent wound closure (p<0.001). Conversely, 50 µM Carbo effectively halted this progression, showing no significant temporal change (p=0.0823). Distinct time-dependent shifts in closure kinetics were also observed across various Carbo+Met, Carbo+BA, and BA+Met combinations (p<0.05), with the magnitude of inhibition varying by concentration. Intergroup analysis confirmed baseline uniformity at 0 h (p>0.05), while treatment-associated differences in wound closure became increasingly evident at 24, 48, and 72 h. Because the scratch assay reflects collective wound closure and may be influenced by both cellular migration and proliferation, these findings were interpreted as treatment-associated alterations in wound-healing behavior rather than as a migration-specific effect (p<0.02).

For CRL-4010 cells (Figure 5B), the control group exhibited expected time-dependent wound closure (p=0.0243). Unlike the tumor line, CRL-4010 cells maintained significant temporal migration changes across all monotherapies (10/50 µM Carbo, 25 mM Met, 50 µM BA; p<0.005) and all evaluated combination groups (p<0.05). While baseline wound areas were uniform at 0 h (p>0.05), intergroup comparisons revealed significant treatment-dependent variations in wound closure kinetics across all subsequent time points (p≤0.0058).

#### Quantitative Analysis of Wound Closure

As shown in Table 1, wound-healing analysis demonstrated that the effects of the different treatment groups on cell migration in the MCF-7 breast cancer cell line varied over time. Compared with the control group, treatment with carboplatin, boric acid, metformin, and their combinations resulted in different degrees of inhibition of wound closure during the 24-, 48-, and 72 h observation periods. Several treatment groups, particularly those containing carboplatin in combination with boric acid or metformin, exhibited significantly reduced wound closure compared with the control, indicating suppression of cell migratory capacity. Statistical analysis also revealed significant differences both within treatment groups over time and among treatment groups at each time point (*p* < 0.05).

As shown in Table 2, wound-healing analysis revealed that the different treatment groups exerted distinct effects on the migratory behavior of CRL-4010 cells throughout the 72 h observation period. Compared with the control group, carboplatin, boric acid, metformin, and their combination treatments produced varying degrees of inhibition of wound closure. Notably, treatment groups containing boric acid alone or in combination with carboplatin exhibited the strongest suppression of wound healing, with complete inhibition of wound closure observed at certain time points. Statistical analysis demonstrated significant differences both within treatment groups over time and among treatment groups at each observation time point (*p* < 0.05), indicating that the treatments differentially affected the migratory capacity of CRL-4010 cells.

### 3.4. Effects of Treatments on Oxidative Stress

**Figure 6:**
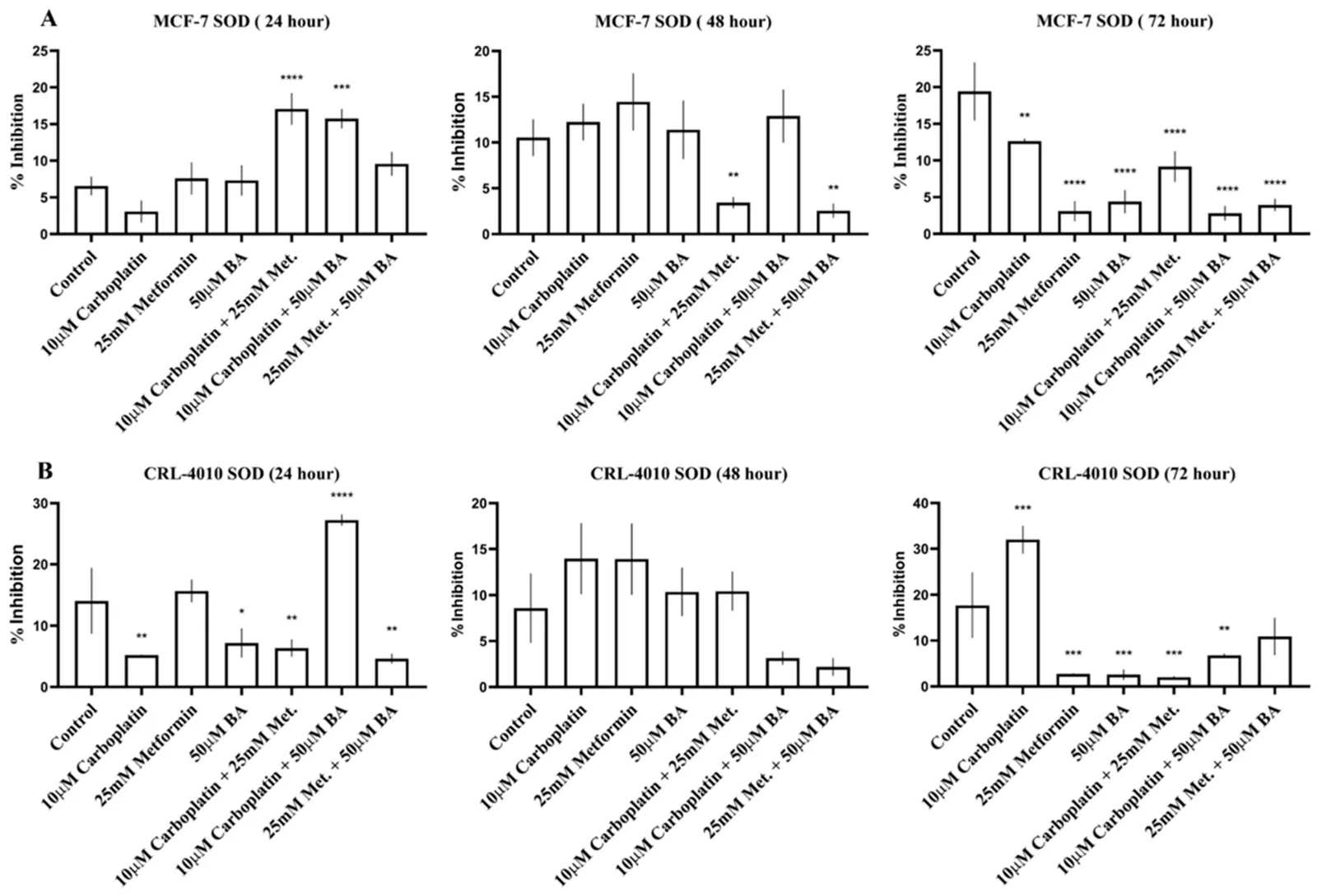
Effects of carboplatin, metformin, boric acid (BA), and their combinations on intracellular Cu, Zn-superoxide dismutase (SOD) activity in MCF-7 and CRL-4010 cells. Intracellular SOD activity was measured after 24, 48, and 72 h of treatment. (**A**) MCF-7 breast cancer cells at 24, 48, and 72 h, respectively. (**B**) CRL-4010 normal mammary epithelial cells at 24, 48, and 72 h, respectively. Data are presented as mean ± standard deviation (SD) from three independent experiments. Statistical significance was determined using one-way analysis of variance (ANOVA) followed by Fisher’s least significant difference (LSD) post hoc test. * *p* < 0.05, ** *p* < 0.01, *** *p* < 0.001, and **** *p* < 0.0001 versus the untreated control.

Figure 6 illustrates the effects of carboplatin, metformin, BA, and their combinations on intracellular superoxide dismutase (SOD) activity in MCF-7 and CRL-4010 cells following 24, 48, and 72 h of treatment. Significant treatment- and time-dependent alterations in SOD activity were observed in both cell lines, indicating modulation of cellular antioxidant defense mechanisms in response to the different treatment regimens.

## 4. Discussion

The present study investigated the effects of carboplatin, metformin, boric acid (BA), and their combinations on cell viability, wound closure, and SOD activity in MCF-7 breast cancer cells and CRL-4010 normal mammary epithelial cells. Combination treatments generally produced greater inhibitory effects than individual treatments, particularly after prolonged exposure. Metformin- and BA-containing combinations were associated with greater reductions in cell viability and wound closure under selected experimental conditions, whereas SOD activity showed treatment-, time-, and cell-line-dependent changes. These findings demonstrate distinct phenotypic and antioxidant enzyme responses to the tested treatments; however, the molecular mechanisms underlying these responses cannot be established from the present experimental data.

Cell viability analyses demonstrated that the effects of carboplatin, metformin, and BA were strongly dependent on treatment duration and combination strategy. Carboplatin monotherapy produced relatively modest effects during the early incubation period, whereas metformin- and BA-containing regimens induced progressively greater reductions in MCF-7 cell viability after 48 and 72 h. Previous studies have shown that metformin can influence cellular energy metabolism and signaling pathways involving AMPK and mTOR. These mechanisms may contribute to its effects in cancer models; however, AMPK phosphorylation, mTOR signaling, and related metabolic pathways were not directly assessed in the present study. Therefore, their contribution to the observed responses remains to be established [22,23,24,25,26,27,28]. Similarly, BA has been reported to influence cancer cell proliferation, oxidative balance, cell-cycle progression, and apoptotic pathways [18,29,30,31,32,33,34].

The translational relevance of the concentrations used in the present study requires careful consideration. In humans, boric acid is rapidly absorbed, undergoes little or no metabolism, and is predominantly eliminated unchanged in the urine, with an elimination half-life of approximately 21 h [35,36]. The 10–50 μM BA range used in the present experiments should therefore be considered primarily an experimental exposure range, because its safety and therapeutic achievability in an anticancer setting have not been established. Likewise, the 25–40 mM metformin concentrations used in vitro are substantially higher than plasma concentrations generally reported during standard therapeutic administration and should not be interpreted as direct equivalents of clinical systemic exposure [37]. In contrast, the carboplatin concentrations used in the present study (10–50 μM) are broadly within the range of plasma exposures reported during clinical carboplatin treatment, with a mean maximum plasma concentration of approximately 38.5 μM reported in a pharmacokinetic study [38]. Accordingly, the translational relevance of the three agents differs considerably, and the present findings should be interpreted as preclinical in vitro observations. Further dose–response, pharmacokinetic, and in vivo studies are required to determine whether comparable biological effects can be achieved at clinically relevant and tolerable exposures.

The relatively greater inhibitory effect observed with carboplatin–metformin combinations is consistent with the possibility that metformin-associated metabolic alterations may influence cellular responses to carboplatin. Previous studies have reported that metformin can alter cellular energy metabolism and mitochondrial function [22,23,26]. However, mitochondrial complex I activity, ATP levels, metabolic flux, and DNA repair capacity were not assessed in the present study; therefore, the contribution of these mechanisms to the observed combination effects remains hypothetical. The greater inhibitory effects observed with selected BA-containing combinations may similarly reflect interactions among treatment-induced cellular stress responses. Although changes in SOD activity were observed, intracellular ROS and other molecular indicators of oxidative stress were not measured. Therefore, the involvement of oxidative stress should be regarded as a possible explanation rather than a demonstrated mechanism. Because formal drug-interaction analyses were not performed, the greater effects observed with selected combinations should not be interpreted as evidence of chemosensitization or synergy.

Evaluation of the CRL-4010 cell line demonstrated that treatment responses differed between malignant and non-malignant mammary epithelial cells. Importantly, the effects observed in CRL-4010 cells indicate that the tested combination regimens cannot be considered exclusively tumor-selective based on the present data. Several metformin- and BA-containing combinations produced substantial reductions in CRL-4010 cell viability, particularly following prolonged exposure, and treatment-associated alterations in wound closure were also observed in the normal mammary epithelial cells. These findings highlight a potential limitation of the therapeutic applicability of the tested regimens and indicate that greater inhibitory activity in MCF-7 cells may be accompanied by effects on non-malignant cells. Because complete concentration–response curves and IC50 values were not established for both cell lines in the present experimental design, a quantitative selectivity index or therapeutic window could not be reliably determined. Future studies should therefore include systematic dose–response analyses in malignant and non-malignant cell models to establish IC50 values and selectivity indices and to identify dose ranges that preferentially affect tumor cells while preserving normal cell viability. Although carboplatin monotherapy exhibited relatively limited cytotoxicity during the early incubation period, prolonged exposure to metformin, BA, and several combination regimens significantly reduced cell viability. These findings indicate that the enhanced biological activity observed with combination treatment is not entirely cancer-specific and emphasize the importance of defining an appropriate therapeutic window.

### 4.1. Effects of Combination Treatments on Cell Wound Closure

Cell wound closure is a phenotypic process influenced by cellular migration and proliferation and is commonly used to evaluate treatment-related changes in collective cell movement. In the present study, wound-healing assays demonstrated that both single-agent and combination treatments significantly reduced wound closure in MCF-7 cells, with the most pronounced effects observed after prolonged exposure. Among the tested regimens, metformin-containing combinations consistently produced greater inhibition of wound closure than individual treatments. These findings are in agreement with previous studies showing that metformin suppresses wound closure and invasion through activation of AMPK, inhibition of mTOR signaling, downregulation of matrix metalloproteinases (MMPs), and attenuation of epithelial–mesenchymal transition (EMT) [27,39,40,41]. However, these pathways were not directly evaluated in the present study and therefore cannot be inferred from the wound-healing findings. Likewise, BA significantly reduced wound closure, particularly at higher concentrations and longer incubation periods, supporting previous evidence that boron compounds may influence cellular motility and stress-related responses [31,42,43].

Combination treatments generally produced stronger inhibitory effects on wound closure than monotherapies. The enhanced inhibition of wound closure observed with selected combination treatments indicates a greater reduction in collective wound-healing capacity compared with some single-agent treatments. Although DNA damage, metabolic stress, and redox alterations could potentially contribute to these effects, these molecular processes were not directly assessed in the present study. Thus, the observed changes in wound closure should be interpreted primarily as phenotypic responses rather than direct evidence of specific molecular mechanisms [12,44]. Furthermore, because wound closure may also be influenced by treatment-induced changes in cell proliferation, the present assay cannot distinguish effects on migration from effects related to reduced cell growth. Although wound closure was also reduced in CRL-4010 cells, the magnitude and temporal profile of these changes differed from those observed in MCF-7 cells, indicating different treatment responses between malignant and non-malignant mammary epithelial cells.

### 4.2. Oxidative Stress Responses and SOD Activity

Oxidative stress has a central role in determining both cancer progression and therapeutic response. Reactive oxygen species (ROS) promote genomic instability and tumor progression at moderate levels, whereas excessive ROS accumulation induces oxidative damage and apoptosis. Superoxide dismutase (SOD) constitutes one of the primary intracellular antioxidant defense systems by converting superoxide radicals into hydrogen peroxide, thereby maintaining redox homeostasis. However, changes in SOD activity alone do not establish the presence or magnitude of oxidative stress, particularly in the absence of direct ROS measurements and complementary redox biomarkers [45,46,47].

In the present study, treatment-induced alterations in SOD activity were both time- and cell-line-dependent. In MCF-7 cells, several combination treatments containing metformin or BA were associated with reduced SOD activity after prolonged exposure. This reduction may reflect diminished antioxidant enzyme capacity; however, its biological significance cannot be determined from SOD activity alone. Conversely, carboplatin monotherapy increased SOD activity under selected treatment conditions. Such an increase may represent an adaptive antioxidant response to treatment-related cellular stress, although the absence of direct intracellular ROS measurements prevents definitive interpretation. Similar adaptive increases in antioxidant enzyme activity following platinum-based chemotherapy have previously been described in experimental cancer models [12,32]. Accordingly, the present findings demonstrate treatment-related modulation of SOD activity but do not establish a specific oxidative or redox mechanism.

A different response pattern was observed in CRL-4010 cells. While significant changes in SOD activity were detected during the early and late treatment periods, most treatment groups exhibited relatively stable SOD activity at 48 h, indicating a different temporal pattern of antioxidant enzyme response compared with MCF-7 cells. These findings do not, by themselves, demonstrate either restoration or disruption of overall redox homeostasis.

Collectively, these findings indicate distinct treatment- and cell-line-dependent patterns of SOD activity in malignant and non-malignant mammary cells. MCF-7 cells exhibited sustained reductions in SOD activity following several combination treatments, whereas CRL-4010 cells showed a different temporal response. Importantly, decreased and increased SOD activity should not be interpreted as equivalent biological responses. Reduced activity may reflect diminished antioxidant defense, whereas increased activity may indicate an adaptive antioxidant response; however, SOD activity alone cannot distinguish between these possibilities or determine whether the observed changes are beneficial or detrimental. In the absence of direct ROS measurements and additional oxidative stress biomarkers, the present findings should therefore be interpreted as evidence of altered antioxidant enzyme responses rather than definitive evidence of oxidative damage or sustained oxidative stress. Similarly, the greater effects observed with selected combinations indicate enhanced phenotypic responses but do not establish a specific molecular pathway responsible for these effects. Future studies incorporating direct measurements of ROS, additional antioxidant and oxidative-damage markers, and analyses of relevant metabolic, DNA-damage, and apoptotic pathways are required to clarify the underlying mechanisms.

### 4.3. Clinical Implications

The present in vitro findings indicate that selected combinations of carboplatin with metformin and/or boric acid can produce greater inhibitory effects on cell viability and wound closure than individual treatments under certain experimental conditions. However, these observations should not be interpreted as evidence of clinical therapeutic benefit, drug synergy, or a defined molecular mechanism. Importantly, treatment-related effects were also observed in CRL-4010 cells, indicating that the enhanced inhibitory effects of the combinations were not restricted to malignant cells. In addition, the experimental concentrations of metformin and BA cannot be directly extrapolated to clinically achievable systemic exposures. Further studies incorporating clinically relevant concentration ranges, formal drug-interaction analyses, pharmacokinetic assessment, and in vivo validation are therefore required to determine whether these combinations provide a meaningful therapeutic advantage.

### 4.4. Limitations and Strengths of the Study

Several limitations should be acknowledged. First, this was an in vitro study using a single established breast cancer cell line, and therefore the findings may not fully reflect the complexity of the tumor microenvironment, immune interactions, or pharmacokinetic factors present in vivo. Moreover, reliance on a single cancer cell line limits the generalizability of these results and does not capture the inter-tumoral heterogeneity across breast cancer subtypes. Second, oxidative stress was evaluated solely by measuring SOD activity. SOD activity was not normalized to total protein content or cell number; therefore, treatment-related differences in cell abundance may have influenced the observed values. Additional oxidative stress biomarkers, including intracellular ROS, catalase, glutathione peroxidase, glutathione, malondialdehyde, and total antioxidant capacity, were not assessed. Third, although combination treatments demonstrated enhanced biological effects, formal drug-interaction analyses, such as the Chou–Talalay combination index, were not performed; therefore, synergistic interactions cannot be conclusively established. Furthermore, the tested concentration ranges were insufficient to reliably determine IC50 values for all treatments, precluding calculation of quantitative selectivity indices without extrapolation. The translational relevance of the metformin and BA concentrations is also limited because the experimental concentrations cannot be directly equated with clinically achievable systemic exposures. Finally, the wound-healing assay evaluates collective cell migration but may also be influenced by treatment-induced alterations in cellular proliferation. Future studies incorporating broader dose–response ranges, comprehensive redox profiling, mechanistic analyses, and in vivo validation are warranted.

Despite these limitations, the present study has several strengths. The parallel evaluation of MCF-7 breast cancer cells and CRL-4010 normal mammary epithelial cells under identical experimental conditions enabled direct comparison of treatment responses. In addition, assessment of cell viability, wound closure, and SOD activity across multiple exposure times provided complementary phenotypic and biochemical information. The inclusion of both single-agent and combination regimens further enabled characterization of treatment- and time-dependent response patterns.

## 5. Conclusions

In conclusion, carboplatin, metformin, and boric acid produced treatment- and time-dependent effects on cell viability, wound closure, and SOD activity in MCF-7 breast cancer cells and CRL-4010 normal mammary epithelial cells. Selected combination treatments produced greater inhibitory effects on cell viability and collective wound closure than individual treatments under specific experimental conditions. However, treatment-related effects were also observed in non-malignant CRL-4010 cells. Treatment-associated changes in SOD activity indicate altered antioxidant enzyme responses but do not establish oxidative stress or a specific redox mechanism. Because formal drug-interaction analyses and complete dose–response assessments were not performed, synergy and treatment selectivity cannot be established from the present data. The findings provide a basis for further mechanistic investigation of carboplatin-, metformin-, and boric acid-containing combination treatments, including clinically relevant exposure ranges, comprehensive redox profiling, formal drug-interaction assessment, direct assessment of metabolic signaling, DNA damage, and apoptotic pathways, and in vivo validation.

## Figures and Tables

**Table 1:** Comparison of the effects of different treatment groups on wound-healing rates in the MCF-7 breast cancer cell line.

	0 Hour	24 Hour	48 Hour	72 Hour	*p* Value
**CONTROL**	100.0% ± 5.6	100.0% ± 0.4 ^i^	100.0% ± 33.2 ^i^	100.0% ± 16.7 ^i^	**<0.001**
**10 µM Carboplatin**	116.4% ± 8.3	41.3% ± 8.5 ^ii^	134.0% ± 39.3 ^iii^	175.0% ± 30.3 ^iv^	**0.001**
**50 µM Carboplatin**	66.4% ± 2.5	26.9% ± 5.1	150.3% ± 7.8	181.9% ± 30.5	0.082
**25 mM Metformin**	119.3% ± 6.0	70.7% ± 6.6	292.3% ± 38.8	222.1% ± 49.7 ^v^	**0.001**
**50 µM Boric Acid**	119.3% ± 6.7	26.4% ± 2.3 ^vi^	241.3%± 27.1 ^vii^	238.8% ± 32.3 ^viii^	**0.001**
**10 µM Carboplatin + 25 mM Metformin**	128.5% ± 6.4	71.2% ± 7.4	283.7% ± 77.3 ^ix^	-	**0.020**
**10 µM Carboplatin + 50 µm Boric Acid**	120.4% ± 0.9	23.4% ± 3.1 ^b,i^	245.9% ± 20.1 ^i^	196.2% ± 19.5 ^i^	**<0.001**
**50 µM Carboplatin + 25 mM Metformin**	168.6% ± 1.2 ^a^	48.6% ± 12.8 ^x^	307.2% ± 21.2 ^c,xi^	318.4% ± 40.0 ^d,xii^	**0.001**
**50 µM Carboplatin + 50 µM Boric Acid**	118.9% ± 6.1	30.3% ± 8.9 ^xiii^	174.1% ± 42.0 ^xiv^	176.1% ± 14.9 ^xv^	**0.001**
**50 µM Boric Acid** **+25 mM Metformin**	95.9% ± 4.0	75.8% ± 3.6 ^xvi^	510%.0 ± 49.3 ^xvi^	-	**0.005**
***p* value**	**0.0032**	**0.0021**	**0.0024**	**0.0143**	

All data are presented as mean ± standard deviation (SD) from three independent experiments. Values shown in bold represent statistically significant overall differences among treatment groups at the corresponding time point, as determined by one-way ANOVA (*p* < 0.05). a *p *= 0.0077 vs. 50 µm Carbop group; b *p *= 0.0270 vs. control group; c *p *= 0.0122 vs. control group; d *p *= 0.0077 vs. control group; i *p *< 0.001 vs. 0 h; ii *p* vs. 0.0228 h; iii *p *= 0.0008 vs. 0 h; iv *p *= 0.0011 vs. 0 h; v *p *= 0.0065 vs. 0 h; vi *p *= 0.0003 vs. 0 h; vii *p *= 0.0014 vs. 0 h; viii *p *= 0.0007 vs. 0 h; ix *p *= 0.0257 vs. 24 h; x *p *= 0.0029 vs. 0 h; xi *p *= 0.0023 vs. 0 h; xii *p *= 0.0019 vs. 0 h; xiii *p *= 0.0011 vs. 0 h; xiv *p *= 0.0006 vs. 0 h; xv *p *= 0.0005 vs. 0 h; xvi *p *= 0.0009 vs. 0 h.

**Table 2:** Comparison of wound-healing assay findings in the CRL-4010 cell line.

	0 Hour	24 Hour	48 Hour	72 Hour	*p* Value
**CONTROL**	100.0% ± 1.5	100.0% ± 19.1	100.0% ± 0.0	100.0% ± 22.9	**0.024**
**10 µM Carboplatin**	102.5% ± 5.0	55.6% ± 16.5 ^i^	96% ± 17.6 ^i^	0.0% ± 0.0 ^i^	**<0.001**
**50 µM Carboplatin**	170.1% ± 3.5	106.1% ± 10.2	77.2% ± 0.0 ^ii^	0.0% ± 0.0	**0.001**
**25 mM Metformin**	99.0% ± 2.9	45.5% ± 9.5 ^i^	177.3% ± 34.3 ^i^	154.5% ±10.4 ^iii^	**<0.001**
**50 µM Boric Acid**	103.5% ± 3.9	27.0% ± 4.4 ^iv^	0.0% ± 0.0 ^iv^	0.0% ± 0.0 ^iv^	**<0.001**
**10 µM Carboplatin + 25 mM Metformin**	106.1% ± 5.4	55.8% ± 8.0 ^v^	233.5% ± 20.2 ^v^	151.2% ± 30.6 ^vi^	**0.001**
**10 µM Carboplatin + 50 µm Boric Acid**	94.8% ± 2.6	21.8% ± 5.9 ^iv^	0.0% ± 0.0 ^iv^	0.0% ± 0.0 ^iv^	**<0.001**
**50 µM Carboplatin + 25 mM Metformin**	175.1% ± 1.3 ^a^	170.0% ± 22.4 ^d^	480.4% ± 37.7 ^f,g,vii^	203.0% ± 99.0 ^viii^	**0.011**
**50 µM Carboplatin + 50 µM Boric Acid**	162.3% ± 4.5	106.3% ± 6.3	102.1% ± 42.4 ^ix^	87.7% ± 29.0	**0.001**
**50 µM Boric Acid** **+25 mM Metformin**	179.1% ± 6.8 ^b^	174.2% ± 13.7 ^c,e,x^	420.0% ± 43.6 ^xi^	229.9% ± 16.9 ^xii^	**<0.001**
***p* value**	**0.0024**	**0.0012**	**0.0031**	**0.0058**	

All data are presented as mean ± standard deviation (SD) from three independent experiments. Values shown in bold represent statistically significant overall differences among treatment groups at the corresponding time point, as determined by one-way ANOVA (*p* < 0.05). a *p *= 0.0378 vs. 10 µm Carbop + 50 µm BA group; b *p *= 0.0227 vs. 10 µm Carbop + 50 µm BA group; c *p *= 0.00447 vs. 50 µm BA group; d *p *= 0.0320 vs. 10 µm Carbop + 50 µm BA group; e *p *= 0.0191 vs. 10 µm Carbop + 50 µm BA group; f *p *= 0.0262 vs. 50 µm Carbop; g *p *= 0.0262 vs. 50 µm BA group; i *p *< 0.001 vs. 0 h; ii *p* vs. 0.0249 h; iii *p *= 0.0015 vs. 0 h; iv *p *< 0.0001 vs. 0 h; v *p *= 0.0003 vs. 0 h; vi *p *= 0.001 vs. 0 h; vii *p *= 0.031 vs. 0 h; viii *p *= 0.0217 vs. 0 h; ix *p *= 0.0188 vs. 0 h; x *p *= 0.0163 vs. 0 h; xi *p *= 0.0001 vs. 0 h; xii *p *= 0.0002 vs. 0 h.

## Data Availability

The datasets generated and/or analyzed in the current study are available from the corresponding author upon reasonable request. All data supporting the findings of this study are included within the article.
